# Atrial high-rate episodes predict major adverse cardio/cerebrovascular events in patients with cardiac implantable electrical devices

**DOI:** 10.1038/s41598-021-98258-4

**Published:** 2021-09-23

**Authors:** Ju-Yi Chen, Tse-Wei Chen, Wei-Da Lu

**Affiliations:** Division of Cardiology, Department of Internal Medicine, National Cheng Kung University Hospital, College of Medicine, National Cheng Kung University, 138 Sheng-Li Road, Tainan, 704 Taiwan

**Keywords:** Cardiology, Health care

## Abstract

Patients with atrial high-rate episodes (AHRE) have a high risk of neurologic events, although the causal role and optimal cutoff threshold of AHRE for major adverse cardio/cerebrovascular events (MACCE) are unknown. This study aimed to identify independent factors for AHRE and subsequent atrial fibrillation (AF) after documented AHRE. We enrolled 470 consecutive patients undergoing cardiac implantable electrical device (CIED) implantations. The primary endpoint was subsequent MACCE after AHRE ≥ 6 min, 6 h, and 24 h. AHRE was defined as > 175 beats per minute (bpm) (Medtronic®) or > 200 bpm (Biotronik®) lasting ≥ 30 s. Multivariate Cox regression analysis with time-dependent covariates was used to determine variables associated with independent risk of MACCE. The patients’ median age was 76 year, and 126 patients (26.8%) developed AHRE ≥ 6 min, 63 (13.4%) ≥ 6 h, and 39 (8.3%) ≥ 24 h. During follow-up (median: 29 months), 142 MACCE occurred in 123 patients. Optimal AHRE cutoff value was 6 min, with highest Youden index for MACCE. AHRE ≥ 6 min ~ 24 h was independently associated with MACCE and predicted subsequent AF. Male gender, lower body mass index, or BMI, and left atrial diameter were independently associated with AHRE ≥ 6 min ~ 24 h. Patients with CIEDs who develop AHRE ≥ 6 min have an independently increased risk of MACCE. Comprehensive assessment of patients with CIEDs is warranted.

## Introduction

Cardiac implantable electrical devices (CIEDs), including pacemakers, defibrillators, and biventricular pacing devices, are used to monitor atrial tachyarrhythmias. Recent studies^1–4^ have focused on detecting atrial high-rate episodes (AHRE), also called subclinical atrial fibrillation (SCAF), even in asymptomatic patients. The increased risk of major adverse cardiovascular events (MACE), particularly myocardial infarction (MI), has been studied in patients with atrial fibrillation (AF)^5^, but only rarely in those with AHRE. However, a recent study has demonstrated that longer duration of AHRE independently predicted MACE, including MI, cardiac revascularization, ventricular tachycardia/fibrillation, cardiovascular hospitalization, acute heart failure, and cardiovascular death^6^.

New-onset higher AHRE burden was also associated with subsequent risk for heart failure in patients with CIEDs^7^. The occurrence of AHRE is suggested to be a marker of increased risk of ventricular tachyarrhythmias and poor prognosis^8^. AHRE has also been associated with an increased risk of ischemic stroke and transient ischemic attacks (TIA)^9^; however, this risk seems lower than that in patients with clinical AF^10^. Until now, no study has evaluated the predictive ability of AHRE in association with major adverse cardiovascular and cerebrovascular events (MACCE).

The optimal cutoff value for AHRE duration that will increase risk of MACCE and subsequent AF remains controversial. The current European Society of Cardiology (ESC) guidelines regarding non-valvular AF^11^ state that AHRE > 5–6 min and > 180 bpm increase the risk for ischemic stroke. AHRE lasting ≥ 30 s^12^, ≥ 5 min^13^, ≥ 6 min^3^, ≥ 5.5 h^14^, ≥ 6 h^15^, or ≥ 24 h^16^ are associated with increased risk of stroke. These different cutoff values suggest that patients with implanted CIEDs should undergo regular assessment for detection of AHRE^1–4^, and patients with AHRE should undergo further rhythm assessment (including long-term electrocardiography monitoring) for overt AF and MACCE risk factors.

Reported independent predictors for AHRE occurrence include sick sinus syndrome^17^, increased left atrium (LA) diameter^17,18^, and paced QRS duration^18^. However, a meta-analysis of 28 studies including more than 24,000 patients^19^ revealed that patients’ baseline characteristics were *not* associated with AHRE occurrence, or other factors, including advanced age, lower resting heart rate, diabetes, hypertension, stroke, thromboembolic events, congestive heart failure, increased LA diameter, coronary artery disease, and CHADS_2_ scores. These results suggest that predictors of AHRE are not yet well established, and that additional study is needed to definitively identify independent predictors.

Our study aimed to investigate different cutoff durations of AHRE and incidence rates of MACCE in Taiwanese patients with CIEDs and without a history of AF, and to identify independent predictive factors for AHRE and for subsequent AF after documented AHRE.

## Methods

Consecutive patients aged 18 years or older who underwent CIED implantation (Medtronic®, Minneapolis, MN, USA, or Biotronik®, Lake Oswego, OR, USA): dual chamber pacemaker, dual chamber implantable cardioverter defibrillator, cardiac resynchronization therapy-pacing, and cardiac resynchronization therapy-defibrillator) in the Cardiology Department of the National Cheng Kung University Hospital, Taiwan, from January 2015 to April 2021 were included.

### Ethical considerations

The protocol for this cohort study was reviewed and approved by the Ethics Committee of National Cheng Kung University Hospital, and was conducted according to the guidelines of the International Conference on Harmonization for Good Clinical Practice (B-ER-108-278). All patients provided signed informed consent at the time of their implantation procedures for data to be recorded for later publication.

### Data collection and definitions^4^

Patients’ medical histories and data of co-morbidities and echocardiographic parameters were collected from chart records for retrospective evaluation. Diabetes mellitus was defined by the presence of symptoms and casual plasma glucose concentrations ≥ 200 mg/dL, fasting plasma glucose concentrations ≥ 126 mg/dL, 2-h plasma glucose concentrations ≥ 200 mg/dL from a 75-g oral glucose tolerance test, or when the patient was taking medication for diabetes mellitus. Hypertension was defined as in-office systolic blood pressure values ≥ 140 mmHg and/or diastolic blood pressure values ≥ 90 mmHg, or when the patient was taking antihypertensive medication. Dyslipidemia was defined as: low-density lipoprotein ≥ 140 mg/dL, high-density lipoprotein < 40 mg/dL, triglycerides ≥ 150 mg/dL, or when the patient was taking medication for dyslipidemia. Chronic kidney disease was defined as an estimated glomerular filtration rate (eGFR) < 60 mL/min/1.73 m^2^ for at least 3 months.

The primary endpoint for this study was the occurrence of MACCE after the date of CIED implantation, including ST elevation MI, non-ST elevation MI, unstable angina, systemic thromboembolism, sustained ventricular tachycardia/fibrillation, cerebrovascular events, including stroke or TIA diagnosed by experienced neurologists, or death (cardiac and non-cardiac).

AHRE were extracted from the devices via telemetry at each office visit (3 ~ 6 months). AHRE electrograms were reviewed by at least one experienced electrophysiologist, who carefully considered the possibility that AHRE included lead noise or artifacts, far-field R-waves, paroxysmal supraventricular tachycardia, and visually confirmed AF in the detected AHRE. Atrial sensitivity was programmed to 0.3 mV with bipolar sensing of Medtronic devices and 0.2 mV with bipolar sensing of Biotronik devices. AHRE was defined as heart rate > 175 bpm (Medtronic) or > 200 bpm (Biotronik) and at least 30 s of atrial tachyarrhythmia recorded by the devices on any day during the study period. In order to evaluate the cutoff threshold for primary endpoints, AHRE was categorized by duration: ≥ 6 min ≥ 6 h and ≥ 24 h. If patients had multiple AHREs, the longest AHRE duration was used for analysis. If a patient’s longest AHRE duration was 8 min, the result was counted as AHRE ≥ 6 min and ≥ 6 h.

### Statistical analysis

Categorical variables are presented as percentages, continuous variables as means, and standard deviations for normally distributed values or medians, and interquartile interval (IQI) for non-normally distributed values. The normal distribution for continuous variables was assessed using the Kolmogorov–Smirnov method. Pearson’s chi-square test or Fisher's exact test was used to determine differences in baseline characteristics for categorical variables, and a two-sample student’s t-test or Mann–Whitney U-test was used to analyze continuous variables. Multivariate Cox regression analysis was used to identify variables associated with MACCE occurrence, reported as hazard ratios (HR) with 95% confidence intervals (CI). If the *p*-value in univariable analysis was < 0.05, the parameter was entered into multivariable analysis. Indicators of AHRE ≥ 6 min, 6 h, and 24 h were determined separately as time-dependent covariates in multivariate Cox proportional hazards regression. Because CHA_2_DS_2_-VASc scores overlapped many factors in univariate analysis, it was used as an independent factor in multivariate Cox regression analysis in model B (Table 3). Multivariate logistic regression analysis was used to identify variables associated with subsequent AF and AHRE occurrence, because we could not confirm the time to subsequent AF and AHRE, reported as hazard ratios (HR) with 95% confidence intervals (CI). If the *p*-value in univariable analysis was < 0.05, the parameter was entered into multivariable logistic analysis. The receiver-operating characteristic (ROC) area under the curve (AUC) of AHRE, and the associated 95% confidence intervals (CI) were evaluated for association with future MACCE and new-onset AF after CIED implantation. The optimal cutoff values with the highest Youden index were chosen based on the results of ROC curve analysis and used to evaluate the associated values of AHRE, in minutes, for determining endpoints. For all comparisons, *p* < 0.05 was considered statistically significant. All data were analyzed using SPSS statistical package version 23.0 (SPSS Inc. Chicago, IL, USA).

## Results

**Figure 1:**
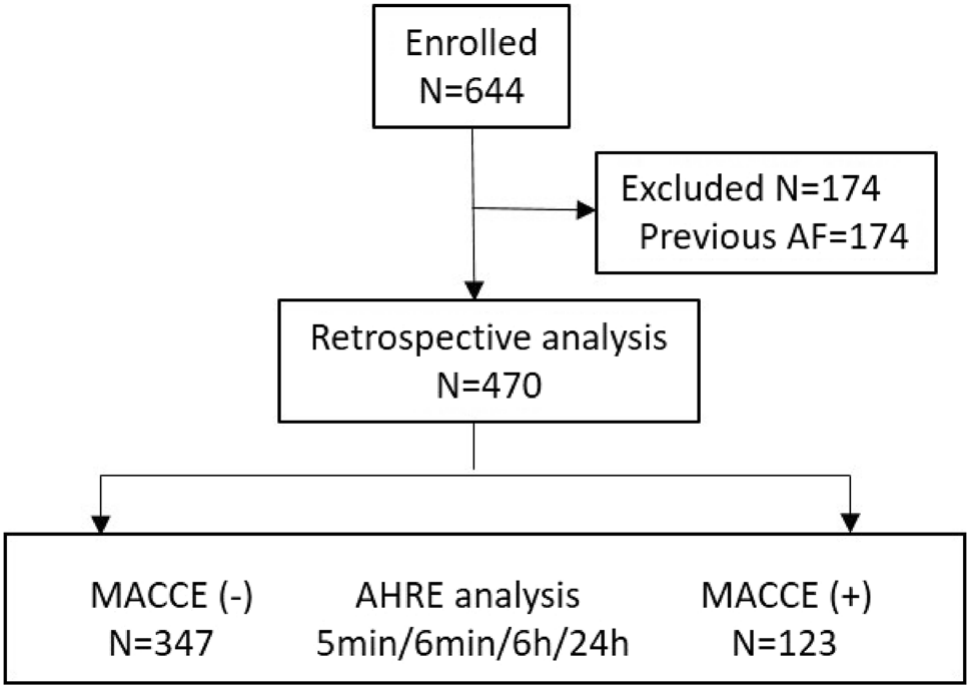
Development of the study cohort. Initially, 644 patients were recruited for the study; however, 174 were excluded due to a previous diagnosis of AF. Therefore, the final study cohort consisted of 470 patients, of which 123 experienced MACCE (*AF *atrial fibrillation, *AHRE* atrial high-rate episodes, *MACCE* major adverse cardio/cerebrovascular events; *N *number).

Between January 1, 2014 and April 2021, 644 consecutive patients receiving CIED transplantation at National Cheng Kung University Hospital were recruited initially. Patients with previous AF (n = 130) were excluded. The final analysis included data from 470 patients, 123 of whom had experienced MACCE (Fig. 1).

The median follow-up period was 29 months after implantation of CIEDs. Table 1 shows patients’ baseline demographic and clinical characteristics based on AHRE durations ≥ 5 min, 6 min, 6 h, and 24 h. Patients’ median age was 76 years, and 58.7% of patients were males. Types of CIEDs included dual chamber pacemaker (376, 80.0%), dual chamber ICD (66, 14.0%), CRTP (23, 4.9%) and CRTD (5, 1.1%). Most CIEDs were Medtronic products (314, 66.8%). The most common indication for CIED implantation was sick sinus syndrome (52.8%), followed by atrioventricular block (27.2%) (Table 1). Overall atrial pacing median percentages (34.0%) and ventricular pacing median percentages (3.1%) were noted. High percentages of hypertension (82.6%), hyperlipidemia (77.9%), and diabetes mellitus (47.9%) suggested the relatively high risk of primary endpoints for the entire study cohort. During follow-up, 126 patients (26.8%) developed AHRE ≥ 6 min; 63 (13.4%) developed AHRE ≥ 6 h; and 39 (8.3%) developed AHRE ≥ 24 h. Components of primary endpoints, including MACCE, time to primary endpoints, incidence rates, and distribution of MACCE, are reported in Table 2. Overall, the follow-up duration represented 1406.08 patient-years of observation, and the total number of MACCE was 142 (incidence rate (IR) 10.10/100 patient-years, 95% CI 5.53–58.48) (Table 2). The most common endpoints were non-cardiac death and acute coronary syndrome (Table 2).

**Table 1:** Baseline characteristics of the overall study group.

Variables	All patients (n = 470)	Primary endpoints Major adverse cardio/cerebrovascular events		Univariate *p*-value
		Yes (n = 123)	No (n = 347)	
Age (years)	76 (65–83)	77 (69–84)	74 (63–82)	0.002
**Gender**				0.002
Male	276 (58.7%)	87 (70.7%)	189 (54.5%)	
Female	194 (41.3%)	36 (29.3%)	158 (45.5%)	
BMI^a^ (kg/m^2^)	24.8 (22.6–26.1)	24.6 (22.5–25.9)	24.8 (22.6–26.2)	0.390
**Device company**				0.249
Medtronic	314 (66.8%)	77 (62.6%)	237 (68.3%)	
Biotronik	156 (33.2%)	46 (37.4%)	110 (31.7%)	
**Device type**				0.066
Dual chamber PM	376 (80.0%)	108 (87.8%)	268 (77.2%)	
Dual chamber ICD	66 (14.0%)	12 (9.8%)	54 (15.6%)	
CRTP	23 (4.9%)	2 (1.8%)	21 (6.1%)	
CRTD	5 (1.1%)	1 (0.8%)	4 (1.2%)	
**Primary indication**				0.043
Sinus node dysfunction	248 (52.8%)	76 (61.8%)	172 (49.6%)	
Atrioventricular block	128 (27.2%)	32 (26.0%)	96 (27.7%)	
VT/VF	94(20.0%)	15 (12.2%)	79 (22.8%)	
Atrial pacing (%)	34.0 (8.6–73.9)	32.2 (6.8–75.5)	34.0 (9.1–73.9)	0.751
Ventricular pacing (%)	3.1 (0.2–96.9)	12.8 (0.2–96.2)	2.1 (0.2–96.9)	0.253
CHA_2_DS_2_-VASc score	3 (2–4)	4 (3–5)	3 (2–4)	< 0.001
HAS-BLED	2 (1–3)	3 (2–3)	2 (1–3)	< 0.001
Hypertension	388 (82.6%)	113 (91.9%)	275 (79.3%)	0.002
Diabetes mellitus	225 (47.9%)	87 (70.7%)	138 (39.8%)	< 0.001
Hyperlipidemia	366 (77.9%)	115 (93.5%)	25 (72.3%)	< 0.001
Prior stroke	25 (5.3%)	7 (5.7%)	18 (5.2%)	0.831
Prior myocardial infarction	91 (19.4%)	44 (35.8%)	47 (13.5%)	< 0.001
**Heart failure**				< 0.001
Preserved EF^b^	52 (11.1%)	14 (11.4%)	38 (11.0%)	
Reduced EF^b^	90 (19.1%)	40 (32.5%)	50 (14.4%)	
Chronic kidney disease	175 (37.2%)	71 (57.7%)	104 (30.0%)	< 0.001
Chronic liver disease	26 (5.5%)	11 (8.9%)	15 (4.3%)	0.054
**Echo parameters**				
LVEF^c^ (%)	67.0 (56.0–74.0)	63.0 (42.0–74.0)	68.0 (60.0–74.0)	0.018
Mitral E/e’	11.0 (8.7–14.0)	12.0 (10.0–16.0)	11.0 (8.2–13.0)	< 0.001
LA^d^ diameter (cm)	3.8 (3.2–4.1)	4.0 (3.5–4.2)	3.6 (3.2–4.1)	0.002
RV^e^ systolic function (s’, m/s)	12.0 (11.0–14.0)	12.0 (10.0–13.0)	12.0 (11.2–14.0)	0.003
**Drug prescribed at baseline**				
Antiplatelets	179 (38.1%)	73 (59.3%)	106 (30.5%)	< 0.001
Anticoagulants	42 (8.9%)	8 (6.5%)	34 (9.8%)	0.271
Beta blockers	164 (34.9%)	47 (38.2%)	117 (33.7%)	0.369
Ivabradine	26 (5.5%)	8 (6.5%)	18 (5.2%)	0.583
Amiodarone	76 (16.2%)	25 (20.3%)	51 (14.7%)	0.145
Dronedarone	5 (1.1%)	3 (2.4%)	2 (0.6%)	0.115
Flecainide	1 (0.2%)	0(0.0%)	1 (0.3%)	1.000
Propafenone	15 (3.2%)	3 (2.4%)	12 (3.5%)	0.769
Digoxin	7 (1.5%)	5 (4.1%)	2 (0.6%)	0.015
Non-DHP CCBs^f^	16 (3.4%)	2 (1.6%)	14 (4.0%)	0.259
RAAS^g^ inhibitors	205 (43.6%)	61 (49.6%)	144 (41.5%)	0.126
Diuretics	78 (16.6%)	31 (25.2%)	47 (13.5%)	0.003
Statins	181 (38.5%)	51 (41.5%)	130 (37.5%)	0.434
Metformin	79 (16.8%)	24 (19.5%)	55 (15.9%)	0.351
SGLT2^h^ inhibitors	15 (3.2%)	7 (5.7%)	8 (2.3%)	0.066
Follow-up duration (months)	29 (14–52)	27 (14–48)	29 (14–53)	0.973
Follow-up times	3 (2–7)	3 (2–6)	3 (2–7)	0.113
AHRE^i^ Duration ≥ 6 min	126 (26.8%)	56 (45.5%)	70 (20.2%)	< 0.001
AHRE^i^ Duration ≥ 6 h	63 (13.4%)	34 (27.6%)	29 (8.4%)	< 0.001
AHRE^i^ Duration ≥ 24 h	39 (8.3%)	21 (17.1%)	18 (5.2%)	< 0.001

Data are presented as medians (interquartile interval) or n (%). Non-parametric continuous variables, as assessed using the Kolmogorov–Smirnov method, were analyzed using the Mann–Whitney U test. Statistical significance is s *p* < 0.05.

^a^BMI, body mass index; ^b^EF, ejection fraction; ^c^LVEF, left ventricular ejection fraction; ^d^LA, left atrium; ^e^RV, right ventricle; ^f^non-DHP CCBs, non-dihydropyridine calcium channel blockers; ^g^RAAS inhibitors SGLT inhibitors; ^h^SGLT2, sodium glucose co-transporters 2; ^i^AHRE, atrial high-rate episodes.

**Table 2:** Types and incidences of major adverse cardio/cerebrovascular events.

Types of MACCE^a^	Number	Incidence rate (100 patient-years)	CI^b^ 95%	Time to event (months)
TIA^c^	12	1.41	0.32–15.32	21.7 ± 25.7 (2–96)
Ischemic stroke	9	1.11	0.37–22.98	20.7 ± 20.6 (1–62)
Embolic event	2	0.39	0.39	13.0 ± 0 (13)
ACS^d^	53	5.24	1.25–135.32	25.8 ± 26.2 (1–108)
Sustained VT^e^/VF^f^	6	0.61	0.37–1.70	25.0 ± 22.6 (9–41)
Cardiac death	1	0.10	0.10	25 ± 0 (25)
Non-cardiac death	59	4.62	0.74–75.32	32.6 ± 30.5 (2–204)
Total events	142	10.10	5.53–58.48	35.9 ± 29.7

Data are presented as mean ± SD or n.

^a^*MACCE* major adverse cardio/cerebrovascular events.

^b^*CI* confidence intervals.

^c^*TIA* transient ischemic attack.

^d^*ACS* acute coronary syndrome: including ST elevation myocardial infarction, non ST elevation myocardial infarction, and unstable angina.

^e^*VT*, ventricular tachycardia.

^f^*VF* ventricular fibrillation.

### Univariate analysis and multivariate Cox regression analysis to identify associations between AHRE durations and MACCE

Univariate analysis revealed that age, male gender, CHA_2_DS_2_-VASc score, HAS-BLED score, the presence of hypertension, diabetes mellitus, hyperlipidemia, prior MI, heart failure with reduced ejection fraction, chronic kidney disease, antiplatelet use, digoxin use, and diuretic use were significantly associated with MACCE occurrence. AHRE lasting ≥ 6 min, ≥ 6 h, and ≥ 24 h were each significantly associated with MACCE (Table 1). Multivariate Cox regression analysis using model A (not including CHA_2_DS_2_-VASc score as a confounder) showed that AHRE ≥ 6 min (HR 1.679, 95% CI 1.147–2.457, *p* = 0.008), and AHRE ≥ 6 h (HR 1.739, 95% CI 1.120–2.701, *p* = 0.014) were each independently associated with MACCE, except in heart failure with reduced ejection fraction, which was also associated with MACCE (Table 3). In model B (which included CHA_2_DS_2_-VASc score as a confounder), AHRE ≥ 6 min (HR 2.254, 95% CI 1.574–3.226, *p* < 0.001), AHRE ≥ 6 h (HR 2.515, 95% CI 1.688–3.746, *p* < 0.001) and AHRE ≥ 24 h (HR 2.185, 95% CI 1.364–3.501, *p* = 0.001) were all independently associated with MACCE, except in the presence of CHA_2_DS_2_-VASc scores.

**Table 3:** Multivariate Cox regression analysis for major adverse cardio/cerebrovascular events.

Variables	Model A-1			Model A-2			Model A-3		
	HR	95% CI	*p*	HR	95% CI	*p*	HR	95% CI	*p*
Age (years)	1.012	0.994–1.031	0.188	1.013	0.995–1.032	0.164	1.012	0.994–1.030	0.200
Gender (male)	0.789	0.526–1.184	0.253	0.787	0.524–1.183	0.250	0.749	0.501–1.120	0.159
Diabetes mellitus (yes)	1.565	0.989–2.474	0.056	1.593	1.006–2.524	0.047	1.580	0.998–2.501	0.051
Hypertension (yes)	0.955	0.443–2.057	0.906	1.022	0.475–2.198	0.956	0.991	0.456–2.155	0.982
Hyperlipidemia (yes)	1.638	0.708–3.789	0.249	1.632	0.708–3.762	0.251	1.693	0.724–3.958	0.224
Prior MI^a^ (yes)	1.447	0.908–2.308	0.121	1.387	0.866–2.221	0.174	1.278	0.794–2.057	0.313
HFrEF^b^ (yes)	2.141	1.278–3.586	0.004	2.188	1.305–3.668	0.003	2.251	1.337–3.790	0.002
CKD^c^ (yes)	1.292	0.838–1.993	0.247	1.219	0.781–1.903	0.383	1.277	0.824–1.979	0.273
Antiplatelet (yes)	1.498	0.984–2.280	0.059	1.534	1.011–2.327	0.044	1.610	1.061–2.444	0.025
Digoxin (yes)	2.351	0.850–6.500	0.099	2.517	0.900–7.041	0.079	2.170	0.784–6.008	0.136
Diuretics (yes)	0.927	0.592–1.1452	0.741	0.866	0.548–1.369	0.537	0.916	0.584–1.437	0.703
AHRE duration ≥ 6 min	1.679	1.147–2.457	0.008						
AHRE duration ≥ 6 h				1.739	1.120–2.701	0.014			
AHRE duration ≥ 24 h							1.603	0.963–2.665	0.069

Variables	Model B-1			Model B-2			Model B-3		
	HR	95% CI	*p*	HR	95% CI	*p*	HR	95% CI	*p*
CHA_2_DS_2_-VASc score	1.680	1.459–1.936	< 0.001	1.666	1.448–1.917	< 0.001	1.645	1.432–1.890	< 0.001
AHRE duration ≥ 6 min	2.254	1.574–3.226	< 0.001						
AHRE duration ≥ 6 h				2.515	1.688–3.746	< 0.001			
AHRE duration ≥ 24 h							2.185	1.364–3.501	0.001

### ROC-AUC determination of AHRE cutoff values as predictive factors for future MACCE

**Figure 2:**
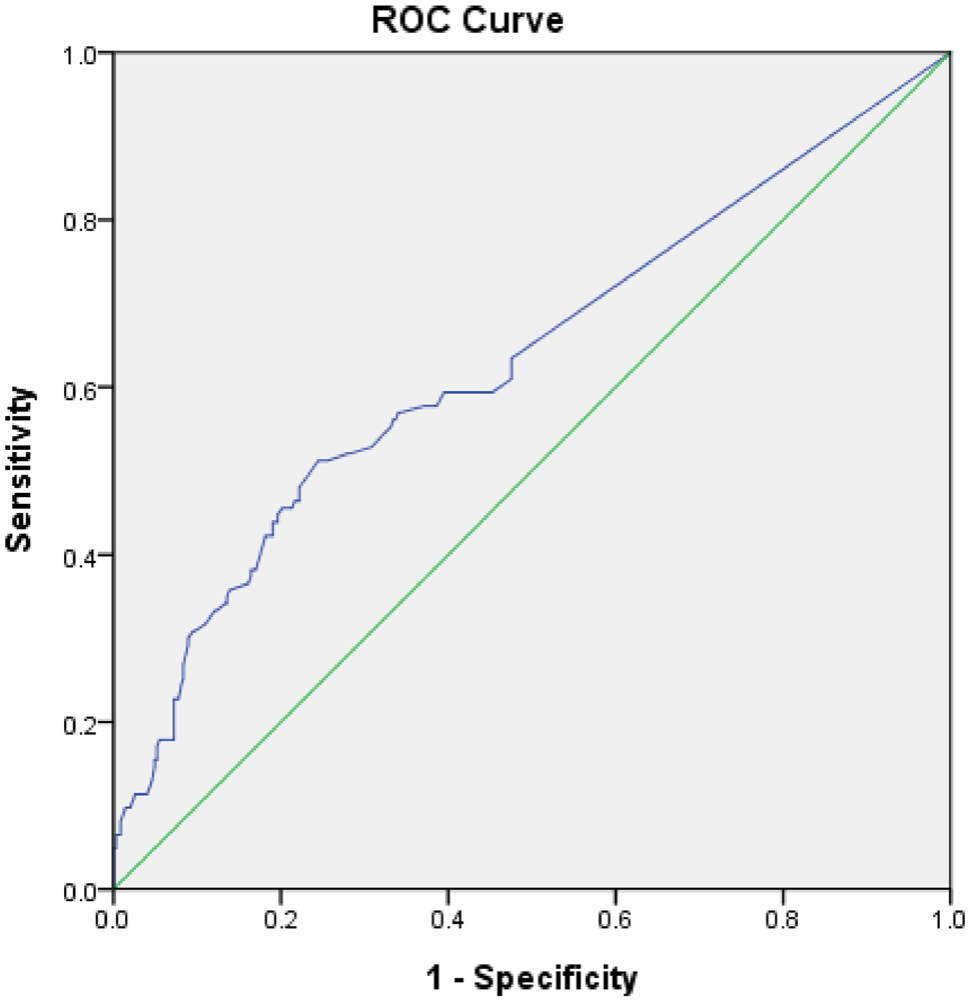
Receiver operating characteristic curve analysis of atrial high-rate episodes (min) in patients with CIEDs with subsequent MACCE. Atrial high-rate episodes (min): cutoff value, 6 min; sensitivity, 45.5%; specificity, 80.0%; 6 h; sensitivity, 28.0%; specificity, 91.5%; 24 h; sensitivity, 17.5%; specificity, 94.5%; area under the curve 0.633; 95% confidence intervals 0.572–0.694; *p* < 0.001.

The optimal AHRE cutoff value predictive of future MACCE was determined to be 6 min, with the highest Youden index (sensitivity, 45.5%; specificity, 80.0%; AUC 0.633; 95% CI 0.572–0.694; *p* < 0.001) (Fig. 2).

### Independent factors for subsequent AF during follow-up

After a median follow-up period of 29 months, 34 patients had newly diagnosed AF. In univariate analysis, male gender, presence of a Medtronic device, sick sinus syndrome, hyperlipidemia, chronic kidney disease, left atrial diameter, HAS-BLED score, and AHRE ≥ 6 min to 24 h were significantly different between patients with or without subsequent AF (Supplementary Table S1). Multivariate logistic regression analysis revealed that AHRE durations ≥ 6 min to 24 h were independently associated with subsequent AF (Table 4). Hazard ratios were increased from AHRE  ≥ 6 min (4.888) to 24 h (5.913), which indicates that longer durations of AHRE pose a higher risk for subsequent AF.

**Table 4:** Multivariate logistic regression analysis for independent factors of subsequent atrial fibrillation.

Variables	Model 1 AHRE ≥ 6 min			Model 2 AHRE ≥ 6 h			Model 3 AHRE ≥ 24 h		
	HR	95% CI	*p*	HR	95% CI	*p*	HR	95% CI	*p*
Male gender	0.426	0.172–1.053	0.065	0.428	0.175–1.048	0.063	0.406	0.164–1.005	0.051
Medtronic device	2.363	0.855–6.527	0.097	2.583	0.948–7.043	0.064	2.450	0.893–6.727	0.082
Sick sinus syndrome	3.951	0.456–34.192	0.212	3.265	0.381–27.816	0.281	3.011	0.352–25.763	0.314
Hyperlipidemia (yes)	2.026	0.231–17.774	0.524	2.666	0.311–22.862	0.371	2.424	0.281–20.924	0.421
Chronic kidney disease (yes)	1.732	0.733–4.096	0.211	1.548	0.662–3.615	0.313	1.514	0.641–3.575	0.345
Left atrial diameter (cm)	1.220	0.636–2.340	0.549	1.271	0.673–2.403	0.460	1.174	0.613–2.248	0.629
HAS-BLED score	1.107	0.711–1.723	0.653	1.067	0.699–1.628	0.765	1.106	0.720–1.698	0.646
AHRE ≥ 6 min	4.888	2.160–11.059	< 0.001						
AHRE ≥ 6 h				3.239	1.419–7.392	0.005			
AHRE ≥ 24 h							5.913	2.451–14.267	< 0.001

### ROC-AUC determination of AHRE cutoff values associated with subsequent AF

**Figure 3:**
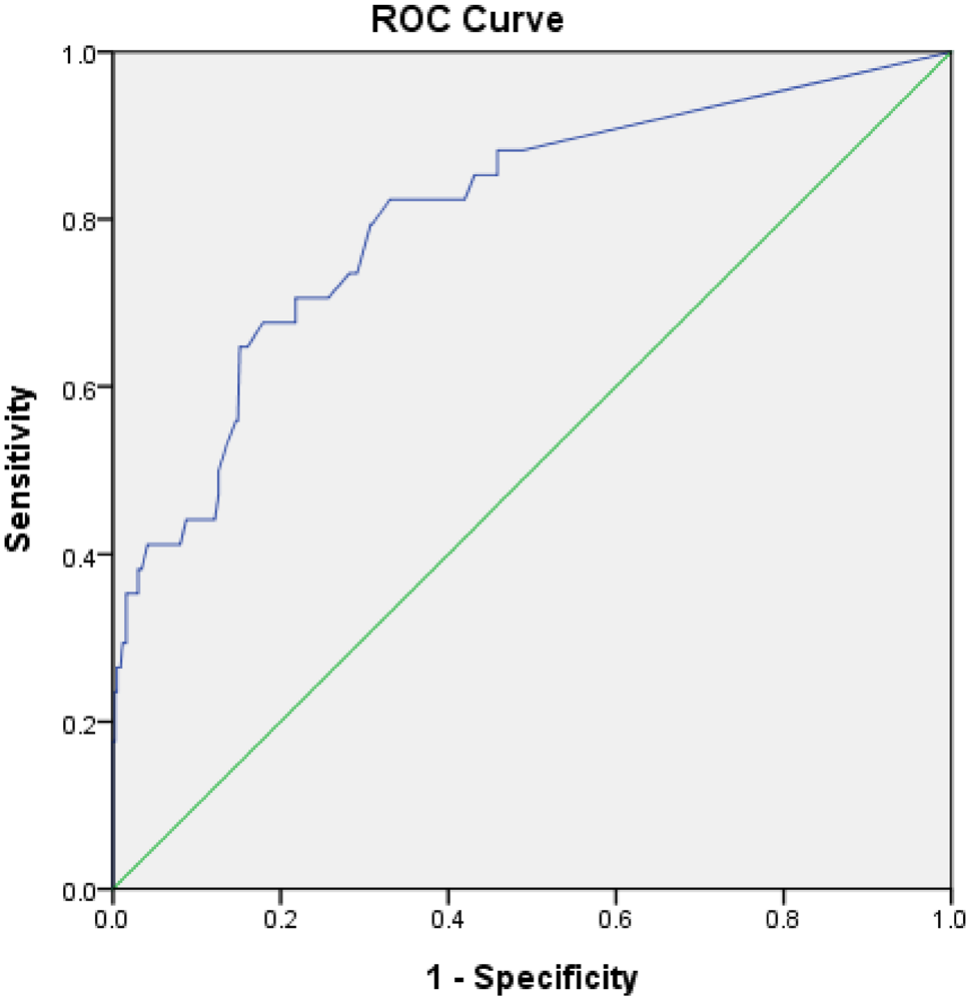
Receiver operating characteristic curve analysis of atrial high-rate episodes (min) for subsequent atrial fibrillation. Atrial high-rate episodes (min): cutoff value, 6 min; sensitivity, 70.6%; specificity, 77.0%; 6 h; sensitivity, 44.1%; specificity, 89.0%; 24 h; sensitivity, 41.2%; specificity, 94.0%; area under the curve 0.806; 95% confidence intervals 0.722–0.889; *p* < 0.001.

The optimal AHRE cutoff value for subsequent AF was determined to be 6 min, with the highest Youden index (sensitivity, 70.6%; specificity, 77.0%; AUC 0.806; 95% CI 0.722–0.889; *p* < 0.001) (Fig. 3).

### Independent factors associated with different AHRE durations

Univariate analysis revealed that male gender, ventricular pacing percentage, hypertension, hyperlipidemia, chronic kidney disease, and left atrial diameter were significantly different between AHRE ≥ 6 min to 24 h (data not shown). Multivariate logistic regression analysis revealed that only left atrial diameter was consistently and independently associated with the occurrences of AHRE ≥ 6 min to 24 h (Table 5).

**Table 5:** Multivariate logistic regression analysis for independent factors of atrial high rate episodes ≥ 6 min, ≥ 6 h, and ≥ 24 h in 470 patients.

Variables	Model 1 AHRE ≥ 6 min			Model 2 AHRE ≥ 6 h			Model 3 AHRE ≥ 24 h		
	HR	95% CI	*p*	HR	95% CI	*p*	HR	95% CI	*p*
Sex (male)	0.646	0.408–1.023	0.063	0.496	0.263–0.934	0.030	0.692	0.327–1.462	0.334
Hypertension (yes)	1.681	0.781–3.621	0.184	1.053	0.405–2.738	0.915	0.969	0.295–3.178	0.958
Hyperlipidemia (yes)	1.974	1.007–3.870	0.048	1.803	0.743–4.377	0.193	3.535	0.957–13.057	0.058
Chronic kidney disease (yes)	1.429	0.907–2.253	0.124	2.104	1.178–3.757	0.012	1.344	0.663–2.723	0.412
Ventricular pacing percentage (%)	1.001	0.996–1.006	0.620	1.000	0.993–1.006	0.937	1.002	0.994–1.009	0.636
Left atrial diameter (cm)	1.472	1.033–2.099	0.032	1.813	1.142–2.879	0.012	2.232	1.254–3.975	0.006

## Discussion

The main finding of this study is that AHRE lasting ≥ 6 min, ≥ 6 h or ≥ 24 h were significantly and independently associated with MACCE in a Taiwanese population with CIEDs and no history of AF. The optimal cutoff value of AHRE for subsequent MACCE and AF was 6 min. Increased LA diameter was independently associated with AHRE duration ≥ 6 min ~ 24 h. These results suggest that early detection of AHRE ≥ 6 min and measurement of LA diameter in patients with CIEDs will allow for early, aggressive therapy to prevent MACCE.

This study was conducted because the optimal cutoff for AHRE duration to predict subsequent MACCE in patients with CIEDs had not been well studied previously and predictive factors were not well established. Sometimes, an exceptionally short duration of atrial tachyarrhythmias may be misclassified as AHRE, due to artifacts and false detection of far-field R-waves by the atrial lead. Current ESC guidelines^11^ recommend that AF can only be diagnosed by 12-lead electrocardiography or by more than 30 s in an ECG strip. The updated ESC guidelines^11^ also recommend that if AHRE ≥ 6 min with high CHA_2_DS_2_-VASc score or AHRE ≥ 24 h occurs, more aggressive monitoring of clinical AF is highly warranted. Although most previous studies have focused on systemic embolic events or neurological events occurring after AHRE, more recent studies have found that MACE, including ventricular tachyarrhythmias^6,8^, heart failure^6^, MI^6^, and cardiovascular death^6^, was also strongly associated with AHRE. Also, the use of different settings for AHRE detection is an important limitation when comparing results between these studies. Vergara et al.^8^ used 200 beats/min as the threshold rate, and Pastori et al.^6^ used 175 beats/min and lasting ≥ 5 min. In the present study, AHRE was defined as heart rate > 175 bpm (Medtronic) or > 200 bpm (Biotronik), and at least 30 s of atrial tachyarrhythmia recorded by the CIEDs on any day during the study period. All study results showed that AHRE was a significant risk factor for future MACE, even with different settings for AHRE detection. Only the present study has demonstrated that AHRE is an independent risk factor for MACCE, and the optimal cutoff value for predicting MACCE is 6 min.

Several pathophysiological mechanisms of MACCE in AHRE have been described, including: (1) AHRE is a precursor of AF, leading to direct coronary or systemic thromboembolism from the left atrium or left atrial appendage; (2) AHRE is associated with multiple atherosclerotic risks and associated inflammatory process, yielding a pro-thrombotic state; and (3) AHRE results in a supply–demand mismatch between the coronary system and heart function^20^.However, while AHRE may not be the only consideration in patients with neurologic events, AHRE duration remains an important area of research for MACCE. Future larger-scale studies are needed to explore AHRE duration cutoffs, with the goal of establishing a standard cutoff for further evaluation of MACCE in patients with AHRE.

The threshold of AHRE for subsequent clinical AF is an important issue in primary care for patients with CIEDs. A recent Japanese study showed that AHRE lasting ≥ 30 s (the shortest AHRE duration reported to date) is a risk factor for ischemic stroke^12^. However, a 5-min cut-off value excludes most misclassified episodes of oversensing or artifacts, and appropriately detects clinical AF^21^. Our study is the first to report that AHRE ≥ 6 min is an independent risk factor for subsequent AF in a Taiwanese population with CIEDs without a history of AF. The percentages of AF occurrence increased as the AHRE duration increased from ≥ 6 min [19.0% (24/126)] to ≥ 24 h [35.9% (14/39)] (data not shown). Early identification of patients with AHRE ≥ 6 min is critical in the clinical detection of AF, and supports a management strategy including early stroke prevention measures.

Awareness of risk factors that contribute to the occurrence of AHRE ≥ 6 min ~ 24 h is clinically relevant to early prevention in CIEDs patients. Previous studies^17–19^ identified several predictors for AHRE, and the common predictive factor in these studies was increased LA diameter^17,18^. A Korean study^17^ demonstrated that LA > 41 mm was significantly associated with occurrence of AHRE ≥ 6 min, and an Indian study reported increased LA diameter contributed to prolonged AHRE^18^. In the present study, increased LA diameter was consistently and significantly associated with AHRE ≥ 6 min to 24 h, comparable to results of the two studies mentioned before^17,18^. Results suggest that, before implantation of CIEDs, evaluation of patients’ echocardiographic parameters must include measurement of LA size, which may lead to early prediction of AHRE ≥ 6 min—a strong predictor for subsequent AF and MACCE.

### Limitations

This study has several limitations. First, this was a single-center, retrospective, observational study with a relatively small number of patients with CIEDs in a hospital setting, and all patients were Taiwanese. As a result, causality cannot be inferred between AHRE and MACCE, and the presence of confounding factors cannot be denied. Also, the results may not be generalizable to other populations. Second, AHRE may have been under- or overestimated due to the default settings in devices designed by different companies, including only Medtronic and Biotronik, used in the present study. Prospective multicenter studies with larger samples are required to confirm the results of this study. Third, this study did not investigate the nature of heart rhythms at the time of the onset of MACCE. Multivariate analysis (model A in Table 3) showed none of the traditional risk factors (DM, HTN, hyperlipidemia, prior MI) were found to be significantly associated with increased MACCE, which left us to wonder whether there was an interaction between these risk factors and AHRE. Finally, in this retrospective analysis of patient data, we could not confirm that patients started anticoagulants due to AHRE detection by the device, although these patients were not excluded because no significant differences were found between anticoagulants use and presence (8, 6.5%) or absence (34, 9.8%) of MACCE (*p* = 0.271), as shown in Table 1.

## Conclusions

MACCE are not uncommon in Taiwanese patients after CIEDs implantation. Episodes of AHRE lasting ≥ 5 min to ≥ 24 h were independent risk factors for MACCE in this population during mid-term follow-up. When AHRE ≥ 5 ~ 6 min is detected in patients with CIEDs, long-term monitoring is advisable, to detect clinical AF as well as to perform comprehensive assessment of MACCE risk using CHA_2_DS_2_-VASc score and echocardiographic study (especially LA size). Our results suggest that early detection of AHRE ≥ 6 min and measurement of LA diameter in patients with CIEDs allows early recognition of the risk of future AF and MACCE. More investigations during early and aggressive antithrombotic therapy in patients with AHRE ≥ 6 min to prevent MACCE are warranted.

### Ethics approval

Approved by the Institutional Review Board of National Cheng Kung University Hospital (B-ER-108-278).

### Consent to participate

All patients provided signed informed consent at the time of CIEDs implantation for their data to be recorded for later publication.

## Supplementary Information

Supplementary Table S1.

## Abbreviations

AF: Atrial fibrillation
AHRE: Atrial high-rate episodes
AMI: Acute myocardial infarction
CIEDs: Cardiac implantable electrical devices
eGFR: Estimated glomerular filtration rate
MACCE: Major adverse cardio/cerebrovascular events
ROC: Receiver-operating characteristic
SCAF: Subclinical atrial fibrillation
TIA: Transient ischemic attacks
