# Supplementary material for: Atrial high-rate episodes predict major adverse cardio/cerebrovascular events in patients with cardiac implantable electrical devices
Source: Sci Rep. 2021 Sep 23;11:18992. doi: 10.1038/s41598-021-98258-4 (PMC8460667; doi:10.1038/s41598-021-98258-4)
Supplement: Supplementary file 1 — Supplementary Table S1. [file 41598_2021_98258_MOESM1_ESM.docx]

**Atrial high-rate episodes predict major adverse cardio/cerebrovascular events in patients with cardiac implantable electrical devices**

Ju-Yi Chen (MD, PhD)*, Tse-Wei Chen (MD), Wei-Da Lu, (MD)

Department of Internal Medicine, National Cheng Kung University Hospital, College of Medicine, National Cheng Kung University, Tainan, Taiwan

| **Supplementary Table S1. Baseline Characteristics of the patients with subsequent atrial fibrillation or not.** | | | | |
| --- | --- | --- | --- | --- |
| **Variables** | **All patients**  **(n=470)** | **Subsequent atrial fibrillation** | | **Univariate**  **P-value** |
|  |  |  |  |  |
|  |  | **Yes**  **(n=34)** | **No**  **(n=436)** |  |
| Age (years) | 76 (65-83) | 77 (68-83) | 75 (65-83) | 0.284 |
| Gender | | | | 0.011 |
| Male | 276 (58.7%) | 27 (79.4%) | 249 (57.1%) |  |
| Female | 194 (41.3%) | 7 (20.6%) | 187 (42.9%) |  |
| BMI^a^ (kg/m^2^) | 24.8 (22.6-26.1) | 24.8 (23.1-26.5) | 24.8 (22.6-26.1) | 0.617 |
| Device company |  |  |  | 0.046 |
| Medtronic | 314 (66.8%) | 28 (82.4%) | 286 (65.6%) |  |
| Biotronik | 156 (33.2%) | 6 (17.6%) | 150 (34.4) |  |
| Device type |  |  |  | 0.067 |
| Dual chamber PM | 376 (80.0%) | 33 (97.1%) | 343 (78.7%) |  |
| Dual chamber ICD | 66 (14.0%) | 0 (0.0%) | 66 (15.1%) |  |
| CRTP | 23 (4.9%) | 1 (2.9%) | 22 (5.0%) |  |
| CRTD | 5 (1.1%) | 0 (0.0%) | 5 (1.1%) |  |
| Primary Indication |  |  |  | 0.001 |
| Sinus node dysfunction | 248 (52.8%) | 21 (61.7%) | 227 (52.1%) |  |
| Atrioventricular block | 128 (27.2%) | 12 (35.3%) | 116 (26.6%) |  |
| VT/VF | 94 (20.0%) | 1 (2.9%) | 93 (21.3%) |  |
| Atrial pacing (%) | 34.0 (8.6-73.9) | 34.9 (8.5-64.6) | 34.0 (8.6-76.3) | 0.877 |
| Ventricular pacing (%) | 3.1 (0.2-96.9) | 13.2 (0.8-43.3) | 3.0 (0.2-97.3) | 0.340 |
| CHA_2_DS_2_-VASc score | 3 (2-4) | 4 (3-4) | 3 (2-4) | 0.297 |
| HAS-BLED | 2 (1-3) | 3 (2-3) | 2 (1-3) | 0.023 |
| Hypertension | 388 (82.6%) | 32 (94.1%) | 356 (81.7%) | 0.096 |
| Diabetes mellitus | 225 (47.9%) | 21 (61.8%) | 204 (46.8%) | 0.092 |
| Hyperlipidemia | 366 (77.9%) | 33 (97.1%) | 333 (76.4%) | 0.002 |
| Prior stroke | 25 (5.3%) | 1 (2.9%) | 24 (5.5%) | 1.000 |
| Prior myocardial infarction | 91 (19.4%) | 9 (26.5%) | 82 (18.8%) | 0.276 |
| Heart failure |  |  |  | 0.296 |
| Preserved EF^b^ | 52 (11.1%) | 6 (17.6%) | 46 (10.6%) |  |
| Reduced EF^b^ | 90 (19.1%) | 8 (23.5%) | 82 (18.8%) |  |
| Chronic kidney disease | 175 (37.2%) | 19 (55.9%) | 156 (35.8%) | 0.020 |
| Chronic liver disease | 26 (5.5%) | 1 (2.9%) | 25 (5.7%) | 0.711 |
| Echo parameters |  |  |  |  |
| LVEF^c^ (%) | 67.0 (56.0-74.0) | 64.5 (52.3-71.5) | 68.0 (56.0-74.0) | 0.216 |
| Mitral E/e’ | 11.0 (8.7-14.0) | 11.9 (9.7-15.3) | 11.0 (8.6-14.0) | 0.259 |
| LA^d^ diameter (cm) | 3.8 (3.2-4.1) | 4.0 (3.5-4.4) | 3.7 (3.2-4.1) | 0.028 |
| RV^e^ systolic function (s’, m/s) | 12.0 (11.0-14.0) | 12.0 (11.0-14.0) | 12.0 (11.0-14.0) | 0.284 |
| Drug prescribed at baseline |  |  |  |  |
| Antiplatelets | 179 (38.1%) | 10 (29.4%) | 169 (38.8%) | 0.280 |
| Anticoagulants | 42 (8.9%) | 17 (50.0%) | 25 (5.7%) | <0.001 |
| Beta blockers | 164(34.9%) | 17 (50.0%) | 147 (33.7%) | 0.055 |
| Ivabradine | 26 (5.5%) | 2 (5.9%) | 24 (5.5%) | 1.000 |
| Amiodarone | 76 (16.2%) | 15(44.1%) | 61 (14.0%) | <0.001 |
| Dronedarone | 5 (1.1%) | 2 (5.9%) | 3 (0.7%) | 0.044 |
| Flecainide | 1(0.2%) | 0 (0.0%) | 1(0.2%) | 1.000 |
| Propafenone | 15 (3.2%) | 3 (8.8%) | 12 (2.8%) | 0.086 |
| Digoxin | 7 (1.5%) | 0 (0.0%) | 7 (1.6%) | 1.000 |
| non-DHP CCBs^f^ | 16 (3.4%) | 1 (2.9%) | 15 (3.4%) | 1.000 |
| RAAS^g^ inhibitors | 205 (43.6%) | 16 (47.1%) | 189 (43.4%) | 0.683 |
| Diuretics | 78 (16.6%) | 9 (26.5%) | 69 (15.8%) | 0.108 |
| Statins | 181(38.5%) | 12 (35.3%) | 169 (38.8%) | 0.689 |
| Metformin | 79 (16.8%) | 8 (23.5%) | 71 (16.3%) | 0.277 |
| SGLT2^h^ inhibitors | 15 (3.2%) | 1 (2.9%) | 14 (3.2%) | 1.000 |
| Follow-up duration (months) | 29 (14-52) | 26 (12-47) | 29 (14-52) | 0.503 |
| Follow-up times | 3 (2-7) | 4 (2-7) | 3 (2-7) | 0.408 |
| AHRE^i^ Duration **≥** 5 min | 129 (27.4%) | 24 (70.6%) | 105 (24.1%) | <0.001 |
| AHRE^i^ Duration **≥** 6 min | 126 (26.8%) | 24 (70.6%) | 102 (23.4%) | <0.001 |
| AHRE^i^ Duration **≥** 6 hr | 63 (13.4%) | 15 (44.1%) | 48 (11.0%) | <0.001 |
| AHRE^i^ Duration **≥** 24 hr | 39 (8.3%) | 14 (41.2%) | 25 (5.7%) | <0.001 |
| Data are presented as medians (interquartile interval) or n (%). Non-parametric continuous variables, as assessed using the Kolmogorov–Smirnov method, were analyzed using the Mann–Whitney U test. Statistical significance is established as p < 0.05.  ^a^BMI, body mass index  ^b^EF, ejection fraction  ^c^LVEF, left ventricular ejection fraction  ^d^LA, left atrium  ^e^RV, right ventricle  ^f^non-DHP CCBs, non-dihydropyridine calcium channel blockers  ^g^RAAS, renin-angiotensin-aldosterone system  ^h^SGLT2, sodium glucose co-transporters 2  ^i^AHRE, atrial high-rate episodes | | | | |
